# Preface

**DOI:** 10.2174/138920292402231013103404

**Published:** 2023-10-27

**Authors:** Fabio Coppede

**Affiliations:** 1 Medical Genetics, Department of Translational Research and of New Surgical and Medical Technologies, University of Pisa, Italy;; 2 Director, Interdepartmental Research Center of Biology and Pathology of Aging, University of Pisa, Italy

Dear authors, readers, and colleagues,

In June 2023, I have been appointed as the new editor-in-chief of *Current Genomics*. The journal has been developed over the last 14 years under the leadership of Prof. Christian Neri, former EiC, whom I would like to thank for his hard work and dedication, which has allowed *Current Genomics* to establish itself as a leading journal in genome sciences. *Current Genomics* publishes high-quality articles in all aspects of genome sciences and aims to keep scientists and researchers updated on genomics discoveries and advancements. I am very honored to take on this role and would like to thank the staff and editorial board members of Bentham Science Publishers for their continued support. I would also like to thank the authors and reviewers of the journal who helped us maintain the high-level quality of published articles.

Enjoy the reading, and best wishes,

